# Characterization of circulating T-, NK-, and NKT cell subsets in patients with colorectal cancer: the peripheral blood immune cell profile

**DOI:** 10.1007/s00262-019-02343-7

**Published:** 2019-05-03

**Authors:** Daniëlle Krijgsman, Natasja L. de Vries, Anni Skovbo, Morten N. Andersen, Marloes Swets, Esther Bastiaannet, Alexander L. Vahrmeijer, Cornelis J. H. van de Velde, Mirjam H. M. Heemskerk, Marianne Hokland, Peter J. K. Kuppen

**Affiliations:** 1 Department of Surgery, Leiden University Medical Center, Albinusdreef 2, 2300 RC Leiden, The Netherlands; 2 Department of Biomedicine, Aarhus University, Aarhus, Denmark; 3 FACS Core Facility, Aarhus University, Aarhus, Denmark; 4 Department of Hematology, Leiden University Medical Center, Leiden, The Netherlands

**Keywords:** Colorectal cancer, Peripheral blood immune cell profile, Cancer immunology, Natural cytotoxicity receptors, Tumor progression, Prognostic biomarkers

## Abstract

**Objective:**

As the development and progression of colorectal cancer (CRC) are known to be affected by the immune system, cell subsets such as T cells, natural killer (NK) cells, and natural killer T (NKT) cells are considered interesting targets for immunotherapy and clinical biomarker research. Until now, the role of systemic immune profiles in tumor progression remains unclear. In this study, we aimed to characterize the immunophenotype of circulating T cells, NK cells, and NKT-like cells in patients with CRC, and to subsequently correlate these immunophenotypes to clinical follow-up data.

**Methods:**

Using multiparameter flow cytometry, the subset distribution and immunophenotype of T cells (CD3^+^CD56^−^), CD56^dim^ NK cells (CD3^−^CD56^dim^), CD56^bright^ NK cells (CD3^−^CD56^bright^), and NKT-like (CD3^+^CD56^+^) cells were investigated in peripheral blood mononuclear cell (PBMC) samples from 71 CRC patients and 19 healthy donors.

**Results:**

CRC patients showed profound differences in immune cell subset distribution and their immunophenotype compared to healthy donors, as characterized by increased percentage of regulatory T cells, and reduced expression level of the natural cytotoxicity receptors NKp44 and NKp46 on both CD56^dim^ NK cells and NKT-like cells. Finally, we showed in a multivariate analysis that above-median percentage of CD16^+^ NKT-like cells was independently associated with shorter disease-free survival in CRC patients.

**Conclusion:**

The altered phenotype of circulating immune cell subsets in CRC and its association with clinical outcome highlight the potential use of PBMC subsets as prognostic biomarkers in CRC, thereby contributing to better insight into the role of systemic immune profiles in tumor progression.

**Electronic supplementary material:**

The online version of this article (10.1007/s00262-019-02343-7) contains supplementary material, which is available to authorized users.

## Introduction

Colorectal cancer (CRC) is a major contributor to cancer-related morbidity and mortality [1]. Approximately one million new cases of CRC arise per year throughout the world, with more than half a million deaths annually [1]. For colorectal tumors that have not spread to distant sites, surgery is the primary treatment [2]. However, around 25% of the patients present with unresectable metastatic disease at the time of diagnosis, and up to 50% percent of early-stage patients develop recurrence or dissemination of the disease following surgery [3]. To reduce the risk of relapse and to prolong the survival of patients with metastatic disease, treatment strategies need to be optimized. Therefore, biomarker application is important since it provides information for therapeutic decision making. As the development and progression of CRC are known to be affected by the immune system, cell subsets such as T cells, natural killer (NK) cells, and natural killer T (NKT) cells are considered interesting targets for immunotherapy and clinical biomarker research. Importantly, the phenotype of circulating lymphocyte subsets may reflect the local immune response in the tumor microenvironment (TME), thereby providing potentially important information regarding disease progression in CRC [4–6].

Different T-cell subsets have been described over the years, each with distinct functions that promote, or inhibit, antitumor immune responses. For instance, cytotoxic (CD8^+^) T cells recognize tumor-associated antigens presented by classical HLA class I molecules. Additionally, CD4^+^ T cells play an important regulatory role via secretion of cytokines and activation of cytotoxic T cells and B cells [7]. Regulatory T cells (T_reg_), a subpopulation of CD4^+^ T cells, have an immunosuppressive function [8]. To escape immune recognition by cytotoxic T cells, tumor cells may downregulate classical HLA class I expression [9]. Studies showed that aberrant HLA class I expression commonly occurs in colorectal tumors [10–14]. These tumors represent potential targets for NK cells [15], which are known to be able to recognize and kill tumor cells with downregulated HLA class I molecules [16, 17].

NK cells can be subdivided based on their CD56 expression: CD56^bright^ NK cells are generally associated with immunoregulatory properties and production of pro-inflammatory cytokines, while CD56^dim^ NK cells primarily exert cytotoxic functions [18, 19]. The NK cell activity is dependent on a delicate balance between activating and inhibitory signals from cell surface receptors [20]. The activating signals are mediated by a wide array of receptors including natural killer group 2-C (NKG2C), natural killer group 2-D (NKG2D), DNAX accessory molecule-1 (DNAM-1), CD161, and natural cytotoxicity receptors (NCRs) NKp30, NKp44, and NKp46. Additionally, CD16 (FcγRIII) on NK cells mediates antibody-dependent cell-mediated cytotoxicity (ADCC) [21]. Furthermore, CD8 is considered a stimulatory cell surface receptor since it enhances the cytolytic activity of NK cells [22]. NK cells also express a range of receptors that provide inhibitory signals upon stimulation. NK cell inhibitory receptors include natural killer group 2-A (NKG2A), and killer cell immunoglobulin (Ig)-like receptors CD158a and CD158b.

Apart from NK- and T cells, peripheral blood comprises other leucocyte subsets with the ability to induce antitumor effects. For instance, NKT cells constitute a unique subset of T cells that lie at the interface between innate and adaptive immunity. Unlike conventional T cells, NKT cells express a T cell receptor that recognizes glycolipids presented by the HLA-like molecule CD1d [23]. Besides, NKT cells express various markers typically associated with NK cells (activating and inhibitory cell-surface receptors) [24]. NKT cells possess cytotoxic capabilities, but are primarily considered to have an important regulatory function via the secretion of large amounts of pro- or anti-inflammatory cytokines upon activation, thereby resulting in amplification or dampening of the immune response [25]. Due to a lack of specific markers, it is not possible as yet to identify the entire NKT cell population using flow cytometry or immunohistochemistry [25]. In many studies, co-expression of CD3 and CD56 is used to identify NKT cells [26–31]. Although it is likely that the CD3^+^CD56^+^ cell population includes “true” CD1d-restricted NKT cells, it has to be taken into account that conventional T cells have also been reported to express CD56 [32, 33]. Thus, since it is unclear whether all CD3^+^CD56^+^ cells are CD1d-restricted, this population is often referred to as “NKT-like”.

In this study, we aimed to characterize the immunophenotype of circulating T cells, NK cells, and NKT-like cells in CRC patients using multiparameter flow cytometry, and to subsequently correlate these immunophenotypes to clinical follow-up data.

## Materials and methods

### Study population

The study population comprised patients diagnosed with CRC who underwent surgical resection of their colorectal tumor at the Leiden University Medical Center (LUMC, the Netherlands) between 2001 and 2007. From these patients, peripheral blood samples were collected within a month prior to surgery. In the present study, patients with histologically proven primary colorectal tumors, Tumor Node Metastasis (TNM) stage 0–IV, surgical R0 resection, and a minimal amount of five million cryopreserved peripheral blood mononuclear cells (PBMCs) were included (*N* = 87). Patients with multiple colorectal tumors at the time of diagnosis or resection were excluded. Patient follow-up was performed until August 2017, with a median follow-up time of 12 years (range 0.1–15.8 years). Peripheral blood samples obtained from 19 healthy donors (Dept. of Hematology, LUMC) served as controls.

### Isolation of peripheral blood mononuclear cells

PBMCs were isolated by Ficoll-Paque (density 1.077g/ml, provided by the apothecary LUMC) density-gradient centrifugation. All PBMC samples were cryopreserved in liquid nitrogen until time of analysis in freezing medium containing 20% heat-inactivated FCS (Thermo Fisher Scientific, Waltham, MA, USA) and 10% DMSO (Avantor, Center Valley, PA, USA). Additionally, PBMCs were isolated from the buffy coat of a healthy donor obtained from the Blood Bank at Aarhus University Hospital (Dept. Clinical Immunology, Aarhus University Hospital, Skejby, Denmark) which was used as an internal control in the flow cytometry experiments. After isolation using Histopaque (Sigma-Aldrich, St. Louis, MO, USA) density-gradient centrifugation, these PBMCs were cryopreserved at − 150 °C until time of use in RPMI-1640 medium (Lonza, Basel, Switzerland) containing 20% heat-inactivated FCS (GE Healthcare, Little Chalfont, United Kingdom), and 10% DMSO (Sigma-Aldrich).

### Flow cytometry antibody staining

Extensive immunophenotyping of circulating T cells, NK cells, and NKT-like cells was performed using multiparameter flow cytometry with directly fluorochrome-conjugated mouse mAb (Supplementary Table 1). Briefly, PBMCs were thawed at 37 °C, washed in PBS (Lonza)/10% FCS (GE Healthcare), and cell count was determined using a NucleoCounter®NC-250™ (ChemoMetec, Allerod, Denmark) according to the manufacturer’s instructions. PBMCs were adjusted to a concentration of 10 × 10^6^ cells/ml and blocked for non-specific Ab binding in PBS/0.5% BSA (Merck Millipore, Billerica, MA, USA)/0.09% sodium azide buffer (Ampliqon, Odense, Denmark) with 5% human AB serum (from a batch of healthy donors obtained from the Blood Bank at Aarhus University Hospital) or 50 μg/ml human Ig (Human Ig, CSL Behring, Bern, Switzerland) for 15 min. Three multicolor flow cytometry panels were designed for the identification of circulating T cells, NK cells, and NKT-like cells (Supplementary Table 1). For each flow panel, a cocktail of Ab was prepared in BD brilliant stain buffer (BD Biosciences) to prevent aggregation of brilliant violet fluorochromes. PBMCs (0.5 × 10^6^ cells) were incubated with the antibody cocktails in the dark for 30 min at room temperature (flow panel 1 and 2) or at 4 °C (flow panel 3), as these conditions provided optimal binding for the included Ab in each panel. To exclude dead cells from the analysis, a live/dead fixable near-infrared dead cell stain kit (Life Technologies, Carlsbad, CA, USA) was included in each staining. After incubation with the Ab cocktails, the cells were washed twice in PBS/0.5% BSA/0.09% sodium azide buffer. Next, PBMCs were fixed in PBS/0.9% formaldehyde (Sigma-Aldrich) before analysis.

### Flow cytometry data analysis

Samples were analyzed immediately after staining on the LSR Fortessa (BD Biosciences) flow cytometer running FACSDiva™ software version 8.0 (BD Biosciences). The data set was analyzed using FlowJo software version 10.1 (Tree Star Inc., Ashland, OR, USA). In each experiment, PBMCs derived from the same buffy coat (described above) were included as an internal control. Instrument performance was verified daily using the Cytometer Setup & Tracking (CS&T) system (BD Biosciences), applying CS&T application settings to ensure comparable flow cytometry results over time. Compensation was carried out with CompBeads (BD Biosciences), OneComp eBeads (eBioscience, Inc., San Diego, CA), CompBeads Plus (BD Biosciences), and ArC reactive beads (Life Technologies) according to the manufacturer’s protocol. The threshold for positive staining was determined using unstained- or fluorescence minus one (FMO) controls when necessary [34, 35]. FMO controls were used for CD56, CD158a, and NKG2A in flow panel 1. Since there was no spectral overlap between the fluorochromes brilliant violet 711 (CD158a) and allophycocyanin (NKG2A), FMO controls for CD158a and NKG2A were combined in a fluorescence minus two (FM2) control following proper verification. Furthermore, an FMO control for CD56 in flow panel 2 was included. To identify circulating T cells, NK cells, and NKT-like cells, a sequential gating strategy for each flow panel was created based on the buffy coat internal control sample (Supplementary Figs. 1, 2, and 3). The expression of phenotypic markers on circulating T cells, NK cells and NKT-like cells was then evaluated by the median fluorescence intensity (MFI) and/or the percentage of positive cells. The markers CD3, CD14, and CD56 were strictly used for gating and not studied in further detail.

### Corrections and statistical analyses

The NKG2A antibody was changed regarding isotype and clone during the flow cytometry experiments due to low quality of a newly ordered Ab batch. The internal control buffy coat was used to correct for differences in the MFI and percentage of positive lymphocytes regarding NKG2A between the two used Ab clones. Statistical analyses were conducted using SPSS statistical software (IBM SPSS Statistics 23, Chicago, USA). The Mann–Whitney *U* test was used to compare the age of the CRC patients with the healthy donors. The sex of CRC patients was compared with healthy donors using a Pearson *χ*^2^ test. Additionally, independent samples *T* tests and Mann–Whitney *U* tests, where appropriate, were used to compare patients with healthy donors and to evaluate differences in patient and tumor characteristics. Furthermore, the Spearman correlation test was used to study the relation of phenotypic markers on different immune cell subsets. Throughout the text, the median percentages or MFI are reported including standard deviations (SD). Kaplan–Meier analyses and log-rank tests were used to investigate and compare survival within patient subgroups. Our primary clinical endpoint was disease-free survival (DFS), which was defined as the time from surgery until recurrence of disease or death, whichever came first, or end of follow-up (censored). Cox regression analysis was used for univariate and multivariate analyses for DFS. We corrected for multiple testing using the Benjamini–Hochberg method, by which adjusted *P *values were calculated (indicated by P*) [36]. *P-* and P* values ≤ 0.05 were considered statistically significant.

## Results

### Patient characteristics

In total, flow cytometry data of 71/87 (81.6%) CRC patients could be included in the analysis. Eight samples were excluded due to low viability of the PBMCs (< 50% viable cells). Additionally, samples from two patients were obtained prior to resection of liver metastases instead of the primary colorectal tumor and, therefore, also excluded from further analysis. Furthermore, four patients were excluded from this study due to a confirmed diagnosis of Lynch syndrome. Finally, two patients were excluded due to pre-surgical chemotherapy before sample collection. Eleven patients included in the study had undergone local radiotherapy prior to the collection of the blood sample. Since local radiotherapy is unlikely to affect the immune system systemically, these patients were not excluded from this study. Table 1 summarizes the clinico-pathological characteristics of the 71 CRC patients included in the analyses, together with 19 healthy donors. When comparing the CRC patients with the healthy donors, no significant difference was observed in relation to sex (Table 1). A trend was observed towards a higher age in the CRC patients compared to the healthy donors, which was not statistically significant (Table 1). Flow cytometry panels 1 and 2 (NK cells and NKT-like cells) were investigated in all included 71 CRC patients and 19 healthy donors. Flow cytometry panel 3 (T cells) was investigated in 47 CRC patients and 10 healthy donors.

**Table 1:** Patient demographics and tumor characteristics

	CRC patients	Healthy donors	
	(*N* = 71)	(*N* = 19)	*P *value
Age*			0.06
Mean (years)	65.6	55.6	
Range	25–85	22–83	
Sex			0.56
Female	32 (45.1%)	10 (52.6%)	
Male	39 (54.9%)	9 (47.4%)	
Tumor location			
Colon	59 (83.1%)		
Rectum	12 (16.9%)		
Tumor stage			
Stage 0	4 (5.6%)		
Stage I	10 (14.1%)		
Stage II	22 (35.2%)		
Stage III	22 (33.8%)		
Stage IV	8 (11.3%)		
Tumor differentiation grade			
Well/moderate	55 (77.5%)		
Poor	13 (18.3%)		
Unknown	3 (4.2%)		
Lymph node invasion			
Yes	30 (56.3%)		
No	40 (42.3%)		
Unknown	1 (1.4%)		
Neoadjuvant radiotherapy			
Yes	10 (14.1%)		
No	61 (85.9%)		
Adjuvant chemotherapy			
Yes	27 (38.0%)		
No	44 (62.0%)		

In this study, the immunophenotype of peripheral blood immune cell profiles was investigated in 71 CRC patients and 19 healthy donors

^*^Age at time of surgery

### Increased percentage of circulating regulatory T cells in colorectal cancer patients compared to healthy donors

**Fig. 1:**
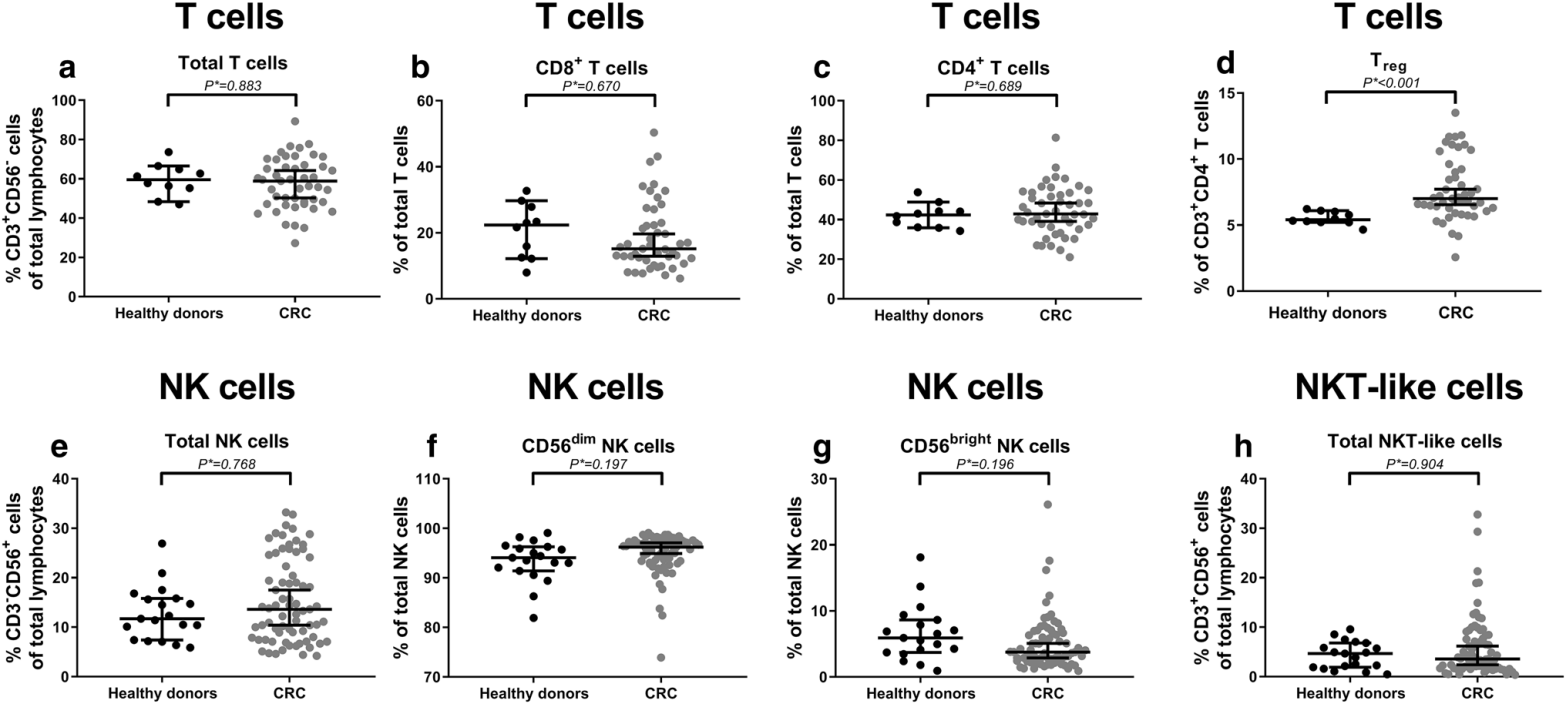
The peripheral blood immune cell subset distribution in CRC patients compared to healthy donors. The distribution of circulating immune cell subsets was compared between CRC patients (grey dots) and healthy donors (black dots). **a** The total percentage of T cells (% CD3^+^CD56^−^ cells of total lymphocytes). **b** Percentage of CD8^+^ T cells (% of total T cells). **c** Percentage of CD4^+^ T cells (% of total T cells). **d** Percentage of T_reg_ (% of CD3^+^CD4^+^ T cells). **e** The total percentage of NK cells (% CD3^−^CD56^+^ cells of total lymphocytes). **f** Percentage of CD56^dim^ NK cells (% of total NK cells). **g** Percentage of CD56^bright^ NK cells (% of total NK cells). **h** The total percentage of NKT-like cells (% CD3^+^CD56^+^ cells of total lymphocytes). The bars show median percentage of the respective immune cell subset including 95% CI

In this study, we compared the immunophenotype of circulating T cells (CD3^+^CD56^−^), NK cells (CD3^−^CD56^+^), and NKT-like cells (CD3^+^CD56^+^) between CRC patients and healthy donors. First of all, the distribution of the different immune cell subsets was studied (Table 2). We hypothesized that CRC patients would present with a general immunocompromised state of circulating immune cells, reflected by a shift in the balance between cytotoxic and regulatory cell subsets. Similar proportions of circulating T cells (P* = 0.883), NK cells (P* = 0.768), and NKT-like cells (P* = 0.904) (% of total lymphocytes) were observed in CRC patients and healthy donors (Fig. 1a, e, h, respectively). Furthermore, no differences were observed in the distribution of circulating CD8^+^ (P* = 0.670) and CD4^+^ (P* = 0.689) T cells (% of T cells) or distribution of circulating CD56^dim^ (P* = 0.197) and CD56^bright^ (P* = 0.196) NK cell populations (% of NK cells) (Fig. 1b, c, f, g, respectively). In contrast, CRC patients presented with an increased percentage of circulating CD127^low^CD25^+^ T_reg_ (P* < 0.001) (% of CD3^+^CD4^+^ T cells) (Fig. 1d) compared to healthy donors.

**Table 2:** Comparison between peripheral blood immune cell profiles in CRC patients and healthy donors

	Healthy controls (*N* = 19)		CRC patients (*N* = 71)			
	Mean	SD	Mean	SD	*P* value	Corrected P* value
Subset distribution						
T cells (%)^*^	59.4	8.2	57.5	13.2	0.669^T^	0.883
*CD8*^*+*^ T cells (%)^*^	20.7	8.3	18.7	10.4	0.378^U^	0.670
*CD4*^*+*^ T cells (%)^*^	42.1	6.1	44.3	12.2	0.400^T^	0.689
*CD127*^*low*^*CD25*^*+*^* T*_*reg*_ (%)^*^	5.5	0.5	7.7	2.4	0.000^U^	**0.000**
*NK cells (%)*	12.8	5.4	15.4	8.5	0.508^U^	0.768
*CD56*^*dim*^* NK cells (%)*	93.4	4.2	95	4.2	0.054^U^	0.197
*CD56*^*bright*^* NK cells (%)*	6.6	4.2	5.1	4.2	0.057^U^	0.196
*NKT-like cells (%)*	4.3	2.8	6.1	6.4	0.656^U^	0.904
CD56^dim^ NK cells						
* CD16* ^*+*^ * (%)*	85.3	6.1	83.7	10.2	0.909^U^	0.989
* CD158a* ^*+*^ * (%)*	35.2	17.1	31.9	16.9	0.477^U^	0.739
* CD158b* ^*+*^ * (%)*	37	11.4	37.5	14.7	0.786^U^	0.937
* NKG2A* ^*+*^ * (%)*	45.6	19.3	43.4	19	0.656^T^	0.884
* NKG2A* ^*+*^ * (MFI)*	6398	2138	7368	2459	0.275^U^	0.533
* NKG2C* ^*+*^ * (%)*	13.7	13	16.2	17.9	0.909^U^	0.972
* NKG2C* ^*+*^ * (MFI)*	2506	2797	2965	3094	0.213^U^	0.440
* CD161 (MFI)*	3111	804	3057	1130	0.816^T^	0.903
* CD8* ^*+*^ * (%)*	34.3	14.3	31.5	14.4	0.467^U^	0.742
* CD8* ^*+*^ * (MFI)*	3481	774	3067	1159	0.067^U^	0.208
* DNAM-1 (MFI)*	560	106	548	146	0.741^T^	0.919
* NKG2D* ^*+*^ * (%)*	89.9	4.4	91.4	4.9	0.134^U^	0.346
* NKG2D* ^*+*^ * (MFI)*	3600	1108	3429	1113	0.554^T^	0.818
* NKp30 (MFI)*	1960	752	1630	900	0.052^U^	0.202
* NKp44* ^*+*^ * (%)*	0.7	0.6	0.6	0.6	0.081^U^	0.239
* NKp44 (MFI)*	131	13	115	26	0.002^U^	**0.041**
* NKp46* ^*+*^ * (%)*	48.7	18	34.7	16.9	0.002^T^	**0.031**
* NKp46 (MFI)*	732	345	478	271	0.003^U^	**0.031**
CD56^bright^ NK cells						
* CD16* ^*+*^ * (%)*	3	2.8	2.5	1.8	0.415^U^	0.695
* CD158a* ^*+*^ * (%)*	6.6	4.2	7	4.8	0.793^U^	0.928
* CD158b* ^*+*^ * (%)*	6.4	3	8.6	5.5	0.097^U^	0.273
* NKG2A* ^*+*^ * (%)*	6.6	4.6	4.5	3.3	0.044^U^	0.210
* NKG2A* ^*+*^ * (MFI)*	17,020	7986	19,052	5829	0.165 ^U^	0.365
* NKG2C* ^*+*^ * (%)*	1.9	2.2	1.2	1.1	0.145^U^	0.333
* NKG2C* ^*+*^ * (MFI)*	1760	967	3038	3831	0.017^U^	0.105
* CD161 (MFI)*	2128	568	1767	578	0.012^U^	0.093
* CD8* ^*+*^ * (%)*	2.4	1.6	1.8	1.7	0.044^U^	0.195
* CD8* ^*+*^ * (MFI)*	2951	886	3098	1318	0.945^U^	0.977
* DNAM-1 (MFI)*	765	130	782	161	0.675^T^	0.872
* NKG2D* ^*+*^ * (%)*	6.2	4.1	4.7	3.7	0.062^U^	0.202
* NKG2D* ^*+*^ * (MFI)*	5321	1354	6210	1837	0.037^U^	0.191
* NKp30 (MFI)*	2145	418	2413	769	0.048^T^	0.198
* NKp44* ^*+*^ * (%)*	2	1.5	1.3	1.2	0.024^U^	0.135
* NKp44 (MFI)*	252	49	236	78	0.099^U^	0.267
* NKp46* ^*+*^ * (%)*	86.1	8.1	80.7	14.3	0.135^U^	0.322
* NKp46 (MFI)*	2343	613	2090	812	0.211^T^	0.451
NKT-like cells						
* CD16* ^*+*^ * (%)*	35.6	17.6	24.6	13.7	0.013^U^	0.090
* CD158a* ^*+*^ * (%)*	12	12.9	9.4	9.8	0.957^U^	0.973
* CD158b* ^*+*^ * (%)*	18.8	19.2	19.2	19.2	0.793^U^	0.910
* NKG2A* ^*+*^ * (%)*	31.9	25.4	28.3	21	0.692^U^	0.876
* NKG2A* ^*+*^ * (MFI)*	4529	4416	3444	3757	0.604^U^	0.851
* NKG2C* ^*+*^ * (%)*	15	15.6	14.2	13.3	0.909^U^	0.955
* NKG2C* ^*+*^ * (MFI)*	2308	2256	3205	3300	0.283^U^	0.532
* CD161 (MFI)*	4509	4407	4445	5434	0.452^U^	0.737
* CD8* ^*+*^ * (%)*	64.1	16.9	70.4	17.9	0.134^U^	0.332
* CD8* ^*+*^ * (MFI)*	11,870	6011	11,673	5556	0.968^U^	0.968
* DNAM-1 (MFI)*	847	211	766	279	0.238^T^	0.476
* NKG2D* ^*+*^ * (%)*	85.4	14.5	88.6	10.7	0.345^U^	0.629
* NKG2D* ^*+*^ * (MFI)*	4730	1589	4995	2041	0.602^T^	0.868
* NKp30 (MFI)*	217	70.1	250	136	0.797^U^	0.898
* NKp44* ^*+*^ * (%)*	1.4	1	1.4	1.2	0.774^U^	0.941
* NKp44 (MFI)*	134	17	120	33	0.002^U^	**0.025**
* NKp46* ^*+*^ * (%)*	5.4	1.9	4.6	2.8	0.011^U^	0.097
* NKp46 (MFI)*	112	53	61	47	0.000^U^	**0.000**

The distribution of circulating immune cell subsets and their immunophenotype was compared between CRC patients (*N* = 71) and healthy donors (*N* = 19). *P* values were corrected for multiple testing using the Benjamini–Hochberg method, by which adjusted *P* values were calculated (indicated by P*). P* values ≤ 0.05 were considered statistically significant and are indicated in bold

^*^T cells were investigated in 10 healthy donors and 47 CRC patients

^T^Independent samples *T* test

^U^Mann-Whitney *U* test

### Reduced expression of natural cytotoxicity receptors on circulating CD56^dim^ NK cells and NKT-like cells from colorectal cancer patients compared to healthy donors

**Fig. 2:**
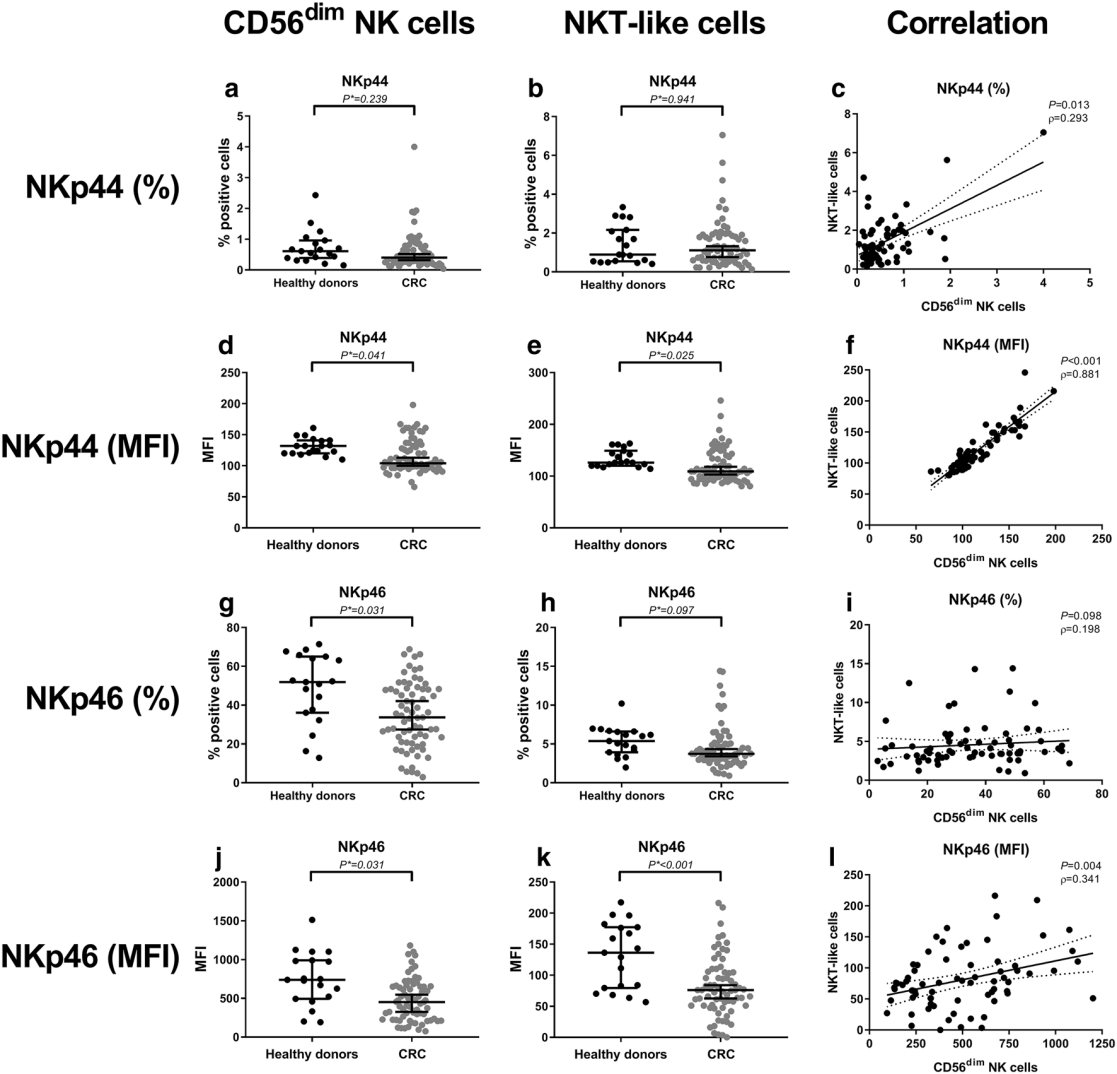
The peripheral blood immunophenotype of CD56^dim^ NK cells and NKT-like cells in CRC patients compared to healthy donors. The peripheral blood immune cell profile was compared between CRC patients (grey dots) and healthy donors (black dots). **a** The percentage of NKp44^+^ CD56^dim^ NK cells. **b** Percentage of NKp44^+^ NKT-like cells. **c** Correlation between the percentage of NKp44^+^ CD56^dim^ NK cells and NKT-like cells. **d** Expression level of NKp44 on CD56^dim^ NK cells. **e** Expression level of NKp44 on NKT-like cells. **f** Correlation between expression level of NKp44 on CD56^dim^ NK cells and NKT-like cells. **g** Percentage of NKp46^+^ CD56^dim^ NK cells. **h** Percentage of NKp46^+^ NKT-like cells. **i** Correlation between the percentage of NKp46^+^ CD56^dim^ NK cells and NKT-like cells. **j** Expression level of NKp46 on CD56^dim^ NK cells **k** Expression level of NKp46 on NKT-like cells. **l** Correlation between expression level of NKp46 on CD56^dim^ NK cells and NKT-like cells. The bars show median percentage or expression level of the respective immunophenotypic marker including 95% CI

We subsequently evaluated the immunophenotype of circulating NK cells and NKT-like cells in CRC patients compared to healthy donors (Table 2). The percentage of NKp44^+^ cells within the CD56^dim^ NK cell subset and NKT-like subset was comparable between CRC patients and healthy donors (P* = 0.239 and P* = 0.941, respectively) (Fig. 2a, b). In contrast, the expression level (MFI) of NKp44 was decreased in CRC patients on both CD56^dim^ NK cells (P* = 0.041) and NKT-like cells (P* = 0.025) compared to healthy donors (Fig. 2d, e). Interestingly, the presence (*P* = 0.013) and expression (*P* < 0.001) of NKp44 on CD56^dim^ NK cells and NKT-like cells strongly correlated (Fig. 2c, f). Hence, patients with a low-percentage positive cells or expression level of NKp44 for CD56^dim^ NK cells also had a low percentage positive cells or expression level of NKp44 for NKT-like cells and the other way around. Additionally, we observed a decreased percentage of NKp46^+^ cells within the CD56^dim^ NK cell subset compared to healthy donors (P* = 0.031) (Fig. 2g), whereas in the NKT-like cell population, no statistically significant difference was found, only a trend (P* = 0.097) (Fig. 2h). In contrast to NKp44, the percentage NKp46^+^ cells did not correlate (only a trend) between CD56^dim^ NK cells and NKT-like cells (*P* = 0.098) (Fig. 2i). Furthermore, the expression level of NKp46 was decreased on both CD56^dim^ NK cells (P* = 0.031) and NKT-like cells (P* < 0.001) in CRC patients compared to healthy donors (Fig. 2j, k). Expression level of NKp46 strongly correlated on CD56^dim^ NK cells and NKT-like cells (*P* = 0.004) (Fig. 2l). In contrast to the CD56^dim^ NK cell and NKT-like cell populations, the percentage of positive cells and expression level of NKp44 and NKp46 were not decreased in the CD56^bright^ NK cell population (Table 2). No differences in other phenotypic markers were observed related to NK cells or NKT-like cells between CRC patients and healthy donors (Table 2).

### No correlation between distribution of immune cell subsets and immunophenotype of circulating lymphocytes and tumor characteristics

After discovering different peripheral blood immune cell profiles in CRC patients compared to healthy donors, the relationship with disease stage was investigated. The course of tumor progression entails a series of stages that ultimately leads to the formation of metastases in distant organs. It is hypothesized that the systemic immune system is activated during tumor progression in CRC upon disruption of the basal membrane located underneath the intestinal epithelium which allows tumor cells to grow into surrounding tissue and to enter blood vessels. As such, CRC patients were subdivided according to their TNM at the time of diagnosis into patients without disruption of the basal membrane (stage 0/I, *N* = 13), and patients with disruption of the basal membrane (stage II/III/IV, *N* = 58).

No differences were observed in the distribution of immune cell subsets or their immunophenotype between CRC patients with or without disruption of the basal membrane. Furthermore, we did not observe any correlation between the immune cell subset distribution or immunophenotype of circulating lymphocytes and tumor location, lymph-node invasion or tumor differentiation grade (data not shown).

### Association between the peripheral blood immune cell profile and disease-free survival of colorectal cancer patients

**Fig. 3:**
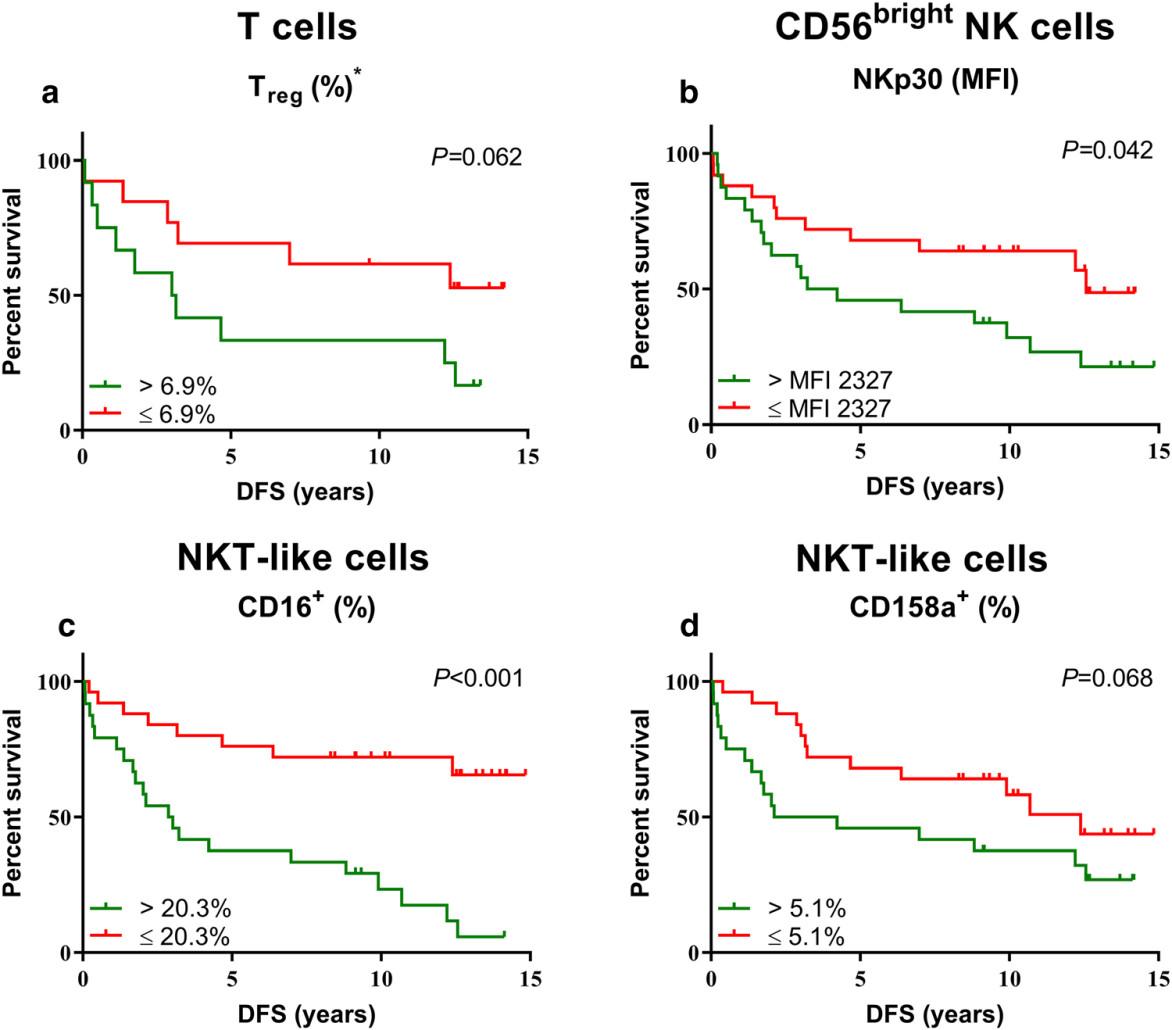
Relationship between the immune subset distribution and immunophenotype of circulating lymphocyte subsets with DFS of CRC patients. DFS curves are shown for stage II and III CRC patients (*N* = 49) at risk for development of metastases. Stratifications were based on the median percentage of positive cells or expression level of the respective immunophenotypic marker. **a** The percentage of T_reg_ (% CD3^+^CD4^+^ T cells). **b** NKp30 expression level on CD56^bright^ NK cells. **c** Percentage of CD16^+^ circulating NKT-like cells and, **d** Percentage of CD158a^+^ NKT-like cells. ^*^This marker was only studied in 25 CRC patients at risk for development of metastasis (stage II and III)

We subsequently wondered whether peripheral blood immune cell profiles were associated with clinical outcome in CRC patients. Therefore, survival plots as well as univariate and multivariate analyses were generated for stage II and III CRC patients at risk of developing metastases. The median percentage of positive cells or expression level of the immunophenotypic marker was used as cutoff. Kaplan–Meier plots and log-rank tests revealed that CRC patients with above-median (> 6.9%, *N* = 12) percentage of circulating T_reg_ showed a trend towards shorter DFS compared to CRC patients with below-median (*N* = 13) percentage (*P* = 0.062) (Fig. 3a), with a hazard ratio (HR) of 2.551 (95% confidence interval (CI) 0.921–7.069, *P* = 0.072). Furthermore, survival analyses revealed that above median (MFI > 2327, *N* = 24) expression of NKp30 on CD56^bright^ NK cells (*P* = 0.042) and above-median percentage ( > 20.3%, *N* = 24) of CD16^+^ NKT-like cells (*P* < 0.001) were associated with short DFS in CRC patients, with a HR of 2.143 (95% CI 1.009–4.551, *P* = 0.047) and 4.697 (95% CI 2.046–10.783, *P* < 0.001), respectively (Fig. 3b;3c). In contrast, CRC patients with above-median (> 5.1%, *N* = 24) percentage of inhibitory receptor CD158a^+^ NKT-like cells showed a trend towards shorter DFS compared to patients with below-median (*N* = 25) percentage (*P* = 0.068) (Fig. 3d) with a HR of 1.969 (95% CI 0.937–4.136, *P* = 0.074). A multivariate analysis was performed for DFS in CRC patients which revealed that above-median percentage of activating receptor CD16^+^ NKT-like cells (HR 4.192, 95% CI 1.578–11.137, *P* = 0.004) remained significantly associated with shorter DFS in CRC patients when corrected for age, sex, tumor stage and lymph-node invasion (Table 3). Thus, the presence of CD16 on circulating NKT-like cells may be a novel prognostic biomarker candidate for clinical outcome of CRC patients. No other correlations between immunophenotype and DFS were observed (Supplementary Table 2).

**Table 3:** Univariate and multivariate analyses for DFS of CRC patients

Factor	Univariate analysis			Multivariate analysis**		
	*P* value	HR	95% CI	*P* value	HR	95% CI
Age^*^	0.053	1.036	0.999–1.075			
Sex						
Female		Ref.				
Male	0.085	1.942	0.912–4.136			
Tumor type						
Colon		Ref.				
Rectum	0.571	1.299	0.526–3.211			
Tumor stage						
Stage II		Ref.				
Stage III	**0.006**	2.946	1.354–6.412			
Tumor differentiation grade						
Well/moderate		Ref.				
Poor	0.885	0.924	0.320–2.669			
Lymph node invasion						
No		Ref.				
Yes	**0.010**	2.825	1.289–6.193			
T_reg_ (%)^***^						
≤ 6.9%		Ref.			Ref.	
> 6.9%	0.072	2.551	0.921–7.069	0.119	3.142	0.744–13.273
NKp30 CD56^bright^ NK cells (MFI)						
≤ MFI 2327		Ref.			Ref.	
> MFI 2327	**0.047**	2.143	1.009–4.551	0.169	1.752	0.788–3.893
CD16^+^ NKT-like cells (%)						
≤ 20.3%		Ref.			Ref.	
> 20.3%	** < 0.001**	4.697	2.046–10.783	**0.004**	4.192	1.578–11.137
CD158a^+^ NKT-like cells (%)						
≤ 5.1%		Ref.			Ref.	
> 5.1%	0.074	1.969	0.937–4.136	0.134	1.803	0.834–3.897

Univariate and multivariate analyses for DFS were generated for stage II and III CRC patients (*N* = 49) at risk for development of metastases. The median percentage of positive cells or median expression level was used as a cutoff [≤ median (*N* = 25) and > median (*N* = 24)]. *P* values, HRs, and their CIs were estimated from a Cox proportional hazard regression model, in which the first category was used as reference group. Statistically significant *P* values are indicated in bold

*Ref*. reference group

^*^Age at time of surgery

^**^Corrected for age, sex, tumor stage and lymph node invasion

^***^This marker was only studied in 25 CRC patients at risk for development of metastasis (stage II and III)

## Discussion

Recently, a new scoring system based on the immune profile in the tumor area, the ‘immunoscore’, has shown to be a strong prognostic biomarker in CRC [37–41]. High density of CD3^+^ and CD8^+^ tumor-infiltrating lymphocytes was reported to correlate with good clinical outcome in CRC [37, 39–41]. We hypothesized that the distribution and phenotype of circulating immune cell subsets may reflect the local immune response in the TME, thereby providing potentially important information regarding disease progression and therapeutic decision making in CRC. To elucidate the role of the peripheral blood immune system in tumor progression and metastasis in CRC, this study aimed to characterize the distribution and immunophenotype of circulating T cells, NK cells, and NKT-like cells in CRC patients. These peripheral blood immune cell profiles were subsequently correlated to clinical follow-up data.

We first demonstrated that CRC patients present with alterations in peripheral blood immune subset distribution. We observed a significant increase in the percentage of circulating T_reg_ in CRC patients compared to healthy donors. This observation is in line with other studies [42, 43, 6], and suggests tumor escape in CRC by increasing T_reg_ which are known to suppress the immune system and, thereby, may support tumor progression using a plethora of different mechanisms [44, 45].

Additionally, we demonstrated that CRC patients present with an altered immunophenotype of circulating CD56^dim^ NK cells compared to healthy donors, characterized by reduced expression of the NCRs NKp44 and NKp46. These findings are consistent with previous studies that showed a reduced number of activating receptor (e.g., NKG2D, NKp30, NKp46, and DNAM-1)-positive NK cells in peripheral blood of patients with CRC [4, 5]. Additionally, NK cells with downregulated activating receptors showed impaired IFN-γ secretion and degranulation upon activation, thereby implying impaired function [4]. These studies did, however, not discriminate between the two major subpopulations of NK cells, namely CD56^dim^ and CD56^bright^ NK cells, which are known to differ in phenotype as well as function [18, 19]. Whereas CD56^dim^ NK cells have the potential to kill tumor cells, CD56^bright^ NK cells primarily produce large amounts of cytokines [18, 19]. We showed that circulating CD56^dim^ NK cells were phenotypically altered in CRC patients, whereas CD56^bright^ NK cells were not, suggesting that this NK cell subset, involved in cytokine production, is phenotypically, and thereby probably also functionally, not altered in CRC patients. This implicates different roles of CD56^dim^ and CD56^bright^ NK cells in cancer progression, thereby emphasizing the need to discriminate between these NK cell subsets in future studies. Furthermore, we also showed downregulation of the NCRs NKp44 and NKp46 on circulating NKT-like cells in CRC patients compared to healthy donors. In contrast to NK cells, the immunophenotype of circulating NKT cells (in both healthy donors and cancer patients) is not well defined due to the lack of specific markers to identify this immune cell subset. To our knowledge, only one study reported on the immunophenotype of circulating NKT-like cells in CRC. This study showed significantly lower numbers of circulating NKG2D^+^ NKT-like cells in CRC patients with metastatic disease compared to healthy donors [46]. Our results suggest that presence of a tumor affects expression levels of NKp44 and NKp46 receptors on both CD56^dim^ NK cells and NKT-like cells. Over the years, many studies have focused on the effects of a tumor on NK cell function. For instance, NK cells in the TME adapt to survive hypoxic stress by upregulating hypoxia-inducible factor (HIF)-1α which is associated with impaired upregulation of NKp44 and NKp46 in response to activating cytokines such as IL-2, IL-12, IL-15, and IL-21 [47]. Furthermore, immune cells and fibroblasts in the TME may produce immunosuppressive cytokines and signal molecules that downregulate [48–50] or prevent IL-2-induced upregulation [51, 52] of NCRs on NK cells. Furthermore, tumor cells may induce downregulation of NCRs on NK cells as a response to hypoxic stress via prostaglandin E2 production or lactate release [53, 54]. Finally, tumor cells may alter the function of NK cells via overexpression of NK cell ligands. For instance, overexpression of proliferating cell nuclear antigen (PCNA) by tumor cells impaired NKp44-mediated NK cell attack in an in vitro model [55, 56]. Whether PCNA also actively downregulates the NKp44 receptor on NK cells is not clear and requires additional research. In conclusion, we observed reduced expression of the NCRs NKp44 and NKp46 on circulating CD56^dim^ NK cells and NKT-like cells in CRC patients, which may be caused by the presence of the tumor via various mechanisms, resulting in functional impairment in these subsets, thereby promoting tumor escape.

Furthermore, we addressed the question whether the peripheral blood immune cell profile in CRC patients was related to tumor stage. Although the peripheral blood immune cell profile was clearly altered in CRC patients as compared to healthy controls, no major shift in the distribution of PBMC subsets was observed in relation with tumor stage of CRC patients. Hence, differences in the peripheral blood immune cell profile were mainly related to the fact that these patients had a colorectal tumor rather than to tumor stage. We did, however, observe a relationship between the immune subset distribution and phenotype of circulating PBMCs and clinical outcome. This suggests that circulating immune cell profiles have prognostic value (i.e., biomarker candidates) for the identification of patients with low tumor grades with a high risk of developing metastatic disease. In general, it is hypothesized that decreased percentages of cytotoxic immune cell subsets or expression levels of activating receptors and increased percentages of regulatory immune cell subsets or expression levels of inhibitory receptors represent an immunocompromised state of the immune system and would therefore be correlated with shorter DFS in CRC patients [57, 58, 4]. In line with this hypothesis, our results revealed that CRC patients with high numbers of circulating T_reg_ showed a trend towards shorter DFS compared to patients with low numbers of circulating T_reg_, an association also observed by others. For instance, an association was reported between T_reg_-mediated suppression of tumor-specific CD4^+^ T cells prior to surgery and tumor recurrence in CRC patients at 12 months [59], whereas FOLFIRI (levo-leucovorin, 5-fluorouracil, irinotexan)- bevacizumab therapy of metastatic CRC patients was associated with better clinical outcome, but also with reduced numbers of circulating T_reg_ [60, 61]. Additionally, in line with our hypothesis, patients with a high percentage of inhibitory receptor CD158a^+^ circulating NKT-like cells showed a trend towards shorter DFS. Since the CD158a receptor recognizes HLA class I molecules, it would be interesting to study HLA class I tumor cell expression in primary colorectal tumors to investigate whether there is an association with the presence of circulating CD158a^+^ NKT-like immune cells. In contrast with the hypothesis, we observed a statistically significant association between a high expression level of activating receptor NKp30 on circulating CD56^bright^ NK cells and a high percentage of CD16^+^ circulating NKT-like cells and short DFS in CRC patients. A multivariate analysis revealed that the latter, high percentage of CD16^+^ NKT-like cells, was even independently associated with shorter DFS in CRC patients. From these observations, it might be deduced that the NKp30 and CD16 receptors are activated during the course of tumor cell dissemination. The exact role of the NKp30 and CD16 receptors in the process of dissemination in CRC patients has yet to be elucidated. Possibly, the status of these receptors in patients with poor clinical outcome might affect the tumor immune response, thus acting as an active selection mechanism by which tumor cells escape immune-mediated destruction. In that case, it would, for instance, be likely that ligands for these receptors on tumor cells are downregulated.

In conclusion, we demonstrated profound differences in subset distribution and immunophenotype of PBMCs in CRC patients compared to healthy donors, characterized by an increased percentage of T_reg_, and reduced expression of NCRs NKp44 and NKp46 on both CD56^dim^ NK cells and NKT-like cells. Additionally, a multivariate analysis revealed that above-median percentage of CD16^+^ NKT-like cells was independently associated with shorter DFS in CRC patients. These findings highlight the potential use of circulating lymphocyte subsets as prognostic biomarkers in CRC and contribute to better insight into the role of systemic immune profiles in tumor progression.

### Electronic supplementary material

Below is the link to the electronic supplementary material.

Supplementary file1 (PDF 1573 kb)

## Abbreviations

ADCC: Antibody-dependent cell-mediated cytotoxicity
CI: Confidence interval
CRC: Colorectal cancer
CS&T: Cytometer setup and tracking
DFS: Disease-free survival
DNAM-1: DNAX accessory molecule-1
FM2: Fluorescence minus two
FMO: Fluorescence minus one
HR: Hazard ratio
HIF-1α: Hypoxia-inducible factor (HIF)-1α
Ig: Immunoglobulin
LUMC: Leiden University Medical Center
MFI: Median fluorescence intensity
NCR: Natural cytotoxicity receptor
NK: Natural killer
NKG2A: Natural killer group 2-A
NKG2C: Natural killer group 2-C
NKG2D: Natural killer group 2-D
NKT: Natural killer T
PBMC: Peripheral blood mononuclear cell
PCNA: Proliferating cell nuclear antigen
Treg: Regulatory T cells
SD: Standard deviation
TME: Tumor microenvironment
TNM: Tumor-node metastasis
