# Supplementary material for: Characterization of circulating T-, NK-, and NKT cell subsets in patients with colorectal cancer: the peripheral blood immune cell profile
Source: Cancer Immunol Immunother. 2019 May 3;68(6):1011–24. doi: 10.1007/s00262-019-02343-7 (PMC6529387; doi:10.1007/s00262-019-02343-7)
Supplement: Supplementary file 1 — Supplementary file1 (PDF 1573 kb) [file 262_2019_2343_MOESM1_ESM.pdf]

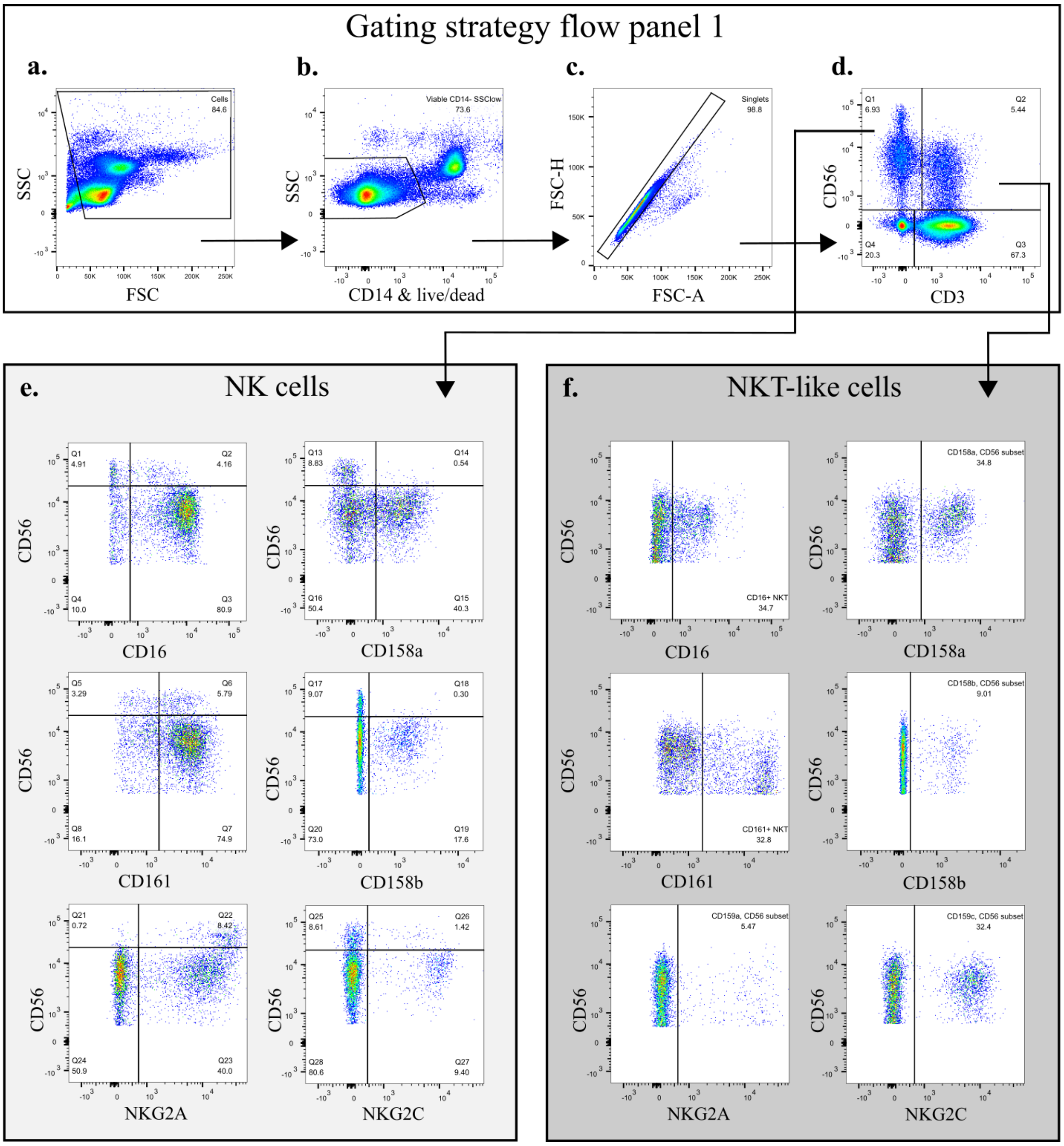

Supplementary Fig.1 *Flow cytometry gating strategy used for the identification of circulating NK cells and NKT-like cells*
Two flow cytometry panels were set up in order to study the immunophenotype circulating NK cells and NKT-like cells. This figure shows the standardized gating strategy used for flow cytometry panel 1. **a.** Mononuclear cells (excluding debris (FSC^{low}/SSC^{low})). **b.** Viable lymphocytes (excluding $CD14^{+}$ monocytes, dead cells, and SSC^{high} cells). **c.** Single lymphocytes (excluding doublets). **d.** NK cells ($CD3^{-}CD56^{+}$ cells) and NKT-like cells ($CD3^{+}CD56^{+}$ cells). Percentage and expression of immunophenotypic markers were determined on the gated $CD56^{dim}$ and $CD56^{bright}$ NK cell populations **e.** and **f.** NKT cell-like population.

Abbreviations: FSC (forward scatter), SSC (side scatter).

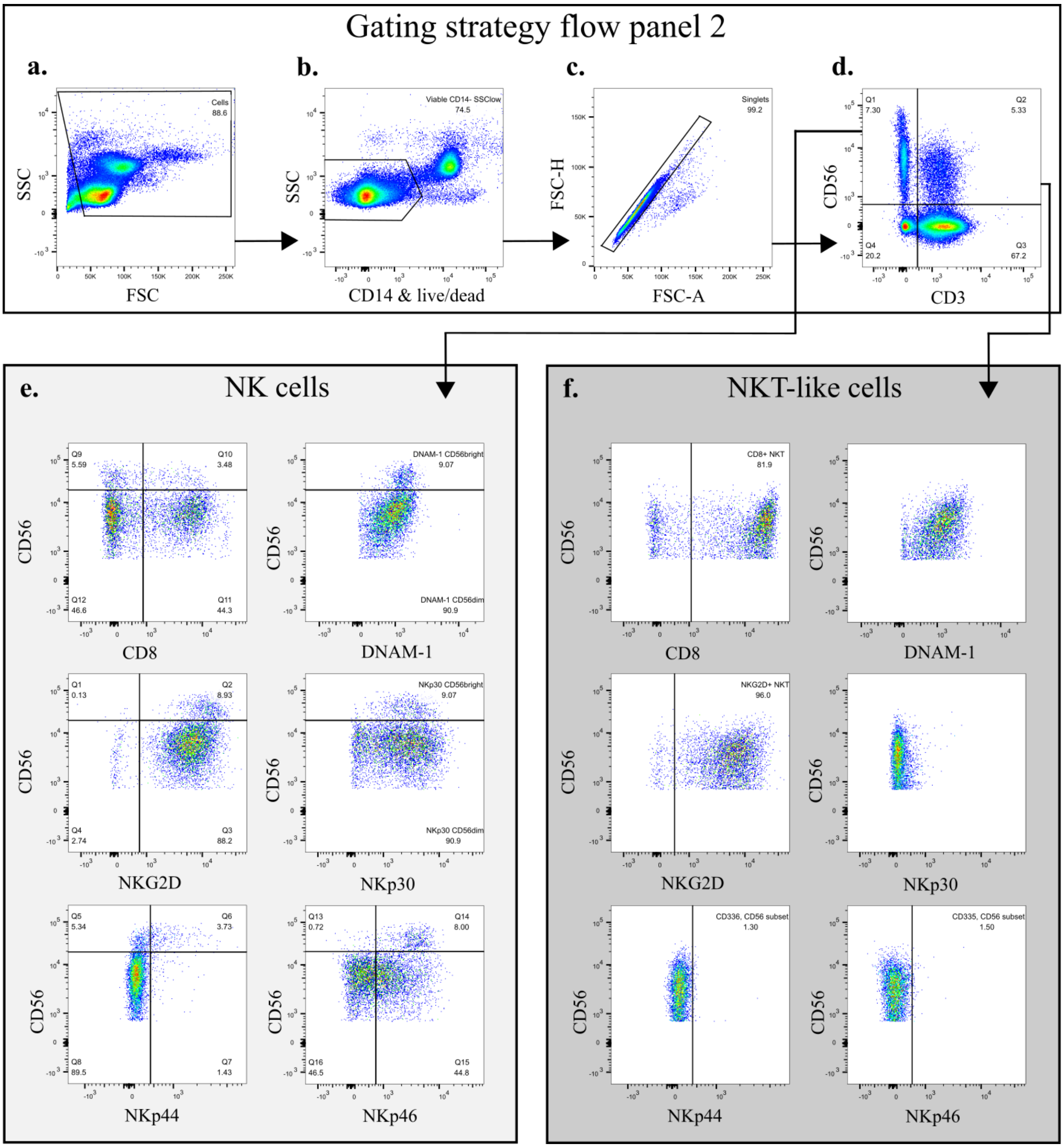

Supplementary Fig.2 *Flow cytometry gating strategy used for the identification of circulating NK cells and NKT-like cells*
Two flow cytometry panels were set up in order to study the immunophenotype circulating NK cells and NKT-like cells. This figure shows the standardized gating strategy used for flow cytometry panel 2. **a.** Mononuclear cells (excluding debris (FSC^{low}/SSC^{low})). **b.** Viable lymphocytes (excluding $CD14^{+}$ monocytes, dead cells, and SSC^{high} cells). **c.** Single lymphocytes (excluding doublets). **d.** NK cells ($CD3^{-}CD56^{+}$ cells) and NKT-like cells ($CD3^{+}CD56^{+}$ cells). Percentage and expression of immunophenotypic markers were determined on the gated $CD56^{dim}$ and $CD56^{bright}$ NK cell populations **e.** and **f.** NKT cell-like population.

Abbreviations: FSC (forward scatter), SSC (side scatter).

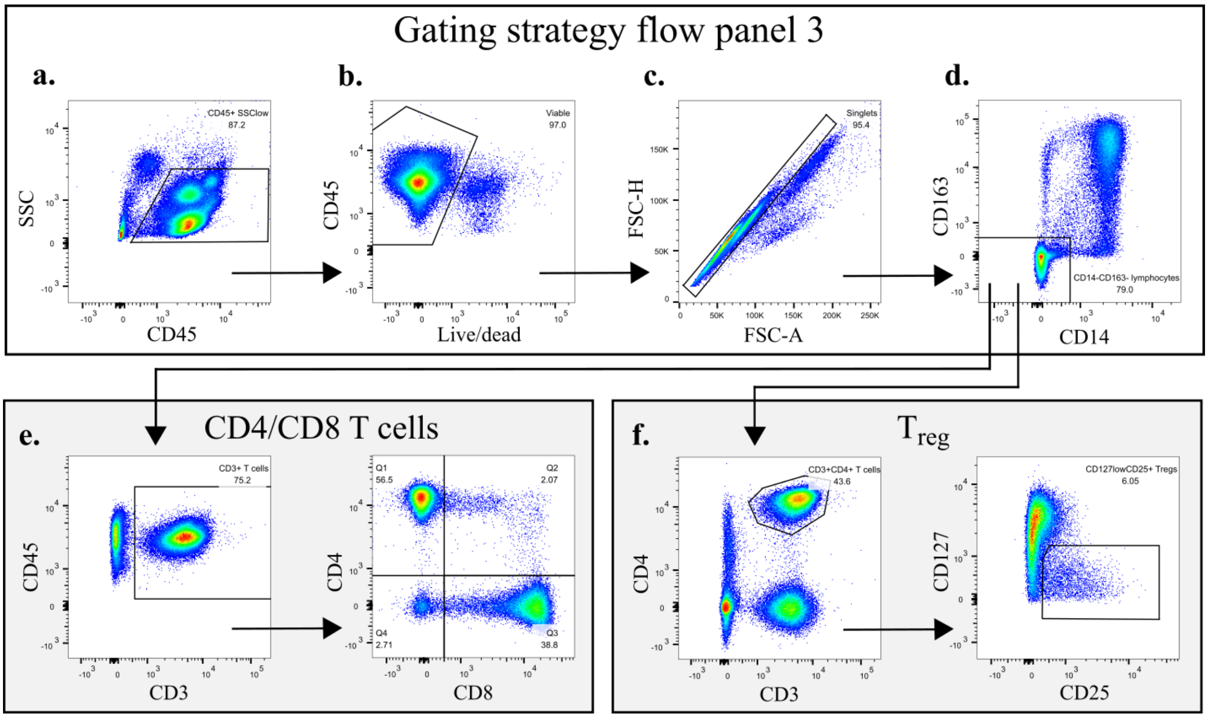

Supplementary Fig.3 *Overview of the flow cytometry gating strategy used for the identification of circulating T cell subsets*
A third flow cytometry panel was set up and a standardized gating strategy was created in order to study the immunophenotype circulating T cell subsets. **a.** Mononuclear cells (excluding $CD45^-$ cells and SSC^{high} cells). **b.** Viable mononuclear cells (excluding dead cells). **c.** Single mononuclear cells (excluding doublets). **d.** Lymphocytes ($CD14^-CD163^-$ cells). **e.** T cells ($CD3^+$), further divided into $CD4^+$ *versus* $CD8^+$ T cells. **f.** Helper T cells ($CD3^+CD4^+$), and their subpopulation T_{reg} ($CD127^{low}CD25^+$ cells).

Abbreviations: FSC (forward scatter), SSC (side scatter)

Supplementary Table 1 *Flow cytometry panels used for the identification of peripheral blood T-, NK-, and NKT-like cell subsets*
Three flow cytometry panels composed of directly fluorochrome-conjugated antibodies against different immunophenotypic markers were designed for the identification of circulating NK- and NKT-like cells (flow panel 1 and 2), and T cell subsets (flow panel 3). The antibodies were purchased from BD Biosciences (San Diego, CA, USA), eBioscience (San Diego, CA, USA), Beckman Coulter (Brea, CA, USA), R&D Systems (Minneapolis, MN, USA), Miltenyi Biotec (Leiden, The Netherlands), Life Technologies (Carlsbad, CA, USA), and Trillium (Brewer, Maine, USA).

Abbreviations: AF (alexa fluor), APC (allophycocyanin), BV (brilliant violet), FITC (fluorescein isothiocyanate), nIR (near-infrared), PE (phycoerythrin), PE-Cy7 (phycoerythrin-cyanine7), PerCP (peridinin chlorophyll protein complex), V500 (violet500).

Flow panel 1 *NK cell and NKT-like cell subsets*				Flow panel 2 *NK cell and NKT-like cell subsets*				Flow panel 3 *T cell subsets*			
Marker	Fluorochrome	Clone	Source	Marker	Fluorochrome	Clone	Source	Marker	Fluorochrome	Clone	Source
CD3	BV605	SK7	BD Biosciences	CD3	BV605	SK7	BD Biosciences	CD3	BV605	SK7	BD Biosciences
CD8	AF700	RPA-T8	BD Biosciences	CD8	AF700	RPA-T8	BD Biosciences	CD4	PE-Cy7	SK3	BD Biosciences
CD14	APC-eFluor780	61D3	eBioscience	CD14	APC-eFluor780	61D3	eBioscience	CD8	AF700	RPA-T8	BD Biosciences
CD16	BV510	3G8	BD Biosciences	CD56	PE-Cy7	N901	Beckman Coulter	CD14	V500	Møp9	BD Biosciences
CD56	PE-Cy7	N901	Beckman Coulter	DNAM-1	FITC	DX11	BD Biosciences	CD16	PerCP-eFluor710	3G8	eBioscience
CD158a	BV711	HP-3E4	BD Biosciences	NKG2D	PE	1D11	eBioscience	CD25	APC	BC96	eBioscience
CD158b	FITC	CH-L	BD Biosciences	NKp46	BV785	9E2/Nkp46	BD Biosciences	CD45	FITC	HI30	BD Biosciences
NKG2A	APC	131411	R&D Systems	NKp44	AF647	P44-8.1	BD Biosciences	CD127	BV421	HIL-7R-M21	BD Biosciences
NKG2A	APC	REA110	Miltenyi Biotec	NKp30	BV421	P30-15	BD Biosciences	CD163	PE	Mac2-158	Trillium
NKG2C	PE	134591	R&D Systems	Live/dead	nIR	-	Life Technologies	Live/dead	nIR	-	Life Technologies
CD161	BV421	DX12	BD Biosciences								
Live/dead	nIR	-	Life Technologies								

Supplementary Table 2 *Survival analyses of CRC patients in relation to immune subset distribution and immunophenotype of circulating lymphocyte subsets*
Survival plots and univariate analyses were generated for stage II and III CRC patients (N=49) at risk of developing metastases. Stratifications were based on the median percentage of positive cells or expression of the respective immunophenotypic marker. The below-median group was used as reference group. *P*-values ≤0.05 were considered statistically significant and indicated in bold.

	Kaplan Meier		Univariate analysis		
	Median	P-value	HR	CI	P-value
**Subset distribution**					
*T cells (%)**	54.6	0.399	0.655	0.243-1.765	0.403
*$CD8^+$ T cells (%)**	16.6	0.222	0.536	0.194-1.481	0.230
*$CD4^+$ T cells (%)**	42.6	0.980	1.013	0.379-2.702	0.980
*$CD127^{low}CD25^+$ T_{reg} (%)**	6.9	0.062	2.551	0.921-7.069	0.072
NK cells (%)	13.3	0.170	1.661	0.798-3.459	0.175
$CD56^{dim}$ NK cells (%)	96.7	0.585	1.226	0.589-2.550	0.586
$CD56^{bright}$ NK cells (%)	3.3	0.380	0.719	0.343-1.507	0.382
NKT-like cells (%)	4.4	0.436	1.335	0.644-2.767	0.438
**$CD56^{dim}$ NK cells**					
$CD16^+$ (%)	83.4	0.412	1.358	0.652-2.830	0.413
$CD158a^+$ (%)	29.9	0.832	0.832	0.443-1.926	0.832
$CD158b^+$ (%)	34.6	0.462	1.316	0.632-2.742	0.463
$NKG2A^+$ (%)	47.2	0.948	0.976	0.470-2.025	0.948
$NKG2A^+$ (MFI)	7212	0.578	0.813	0.391-1.689	0.578
$NKG2C^+$ (%)	12.9	0.559	0.804	0.386-1.675	0.560
$NKG2C^+$ (MFI)	2015	0.605	0.824	0.396-1.716	0.606
CD161 (MFI)	2670	0.606	0.825	0.397-1.716	0.606
$CD8^+$ (%)	25.9	0.645	0.843	0.406-1.749	0.646
$CD8^+$ (MFI)	3091	0.894	1.052	0.500-2.214	0.894
DNAM-1 (MFI)	555	0.464	1.313	0.632-2.725	0.465
$NKG2D^+$ (%)	91.3	0.120	1.784	0.851-3.739	0.125
$NKG2D^+$ (MFI)	3414	0.841	1.078	0.517-2.248	0.841
NKp30 (MFI)	1390	0.180	1.663	0.785-3.525	0.184
$NKp44^+$ (%)	0.5	0.910	1.043	0.500-2.175	0.910
NKp44 (MFI)	111	0.240	1.581	0.732-3.412	0.243
$NKp46^+$ (%)	33.7	0.343	1.434	0.679-3.029	0.345
NKp46 (MFI)	452	0.141	1.768	0.821-3.806	0.146
**$CD56^{bright}$ NK cells**					
$CD16^+$ (%)	1.6	0.358	0.708	0.338-1.484	0.360
$CD158a^+$ (%)	5.2	0.244	1.540	0.741-3.200	0.248
$CD158b^+$ (%)	7.6	0.641	1.189	0.574-2.466	0.641
$NKG2A^+$ (%)	3.2	0.734	0.881	0.423-1.834	0.734
$NKG2A^+$ (MFI)	17899	0.770	1.115	0.537-2.314	0.770
$NKG2C^+$ (%)	0.9	0.414	0.735	0.351-1.541	0.415
$NKG2C^+$ (MFI)	2208	0.303	0.679	0.323-1.426	0.306
CD161 (MFI)	1616	0.683	0.858	0.413-1.786	0.683
$CD8^+$ (%)	1.2	0.655	0.846	0.406-1.761	0.655
$CD8^+$ (MFI)	2897	0.626	1.202	0.573-2.518	0.627
DNAM-1 (MFI)	774	0.232	1.561	0.748-3.259	0.236
$NKG2D^+$ (%)	3.2	0.380	0.719	0.343-1.507	0.382
$NKG2D^+$ (MFI)	6342	0.376	1.394	0.666-2.918	0.378
NKp30 (MFI)	2327	**0.042**	2.143	1.009-4.551	**0.047**
$NKp44^+$ (%)	0.9	0.152	0.581	0.274-1.232	0.157
NKp44 (MFI)	238	0.776	1.112	0.535-2.311	0.776
$NKp46^+$ (%)	85.4	0.287	1.487	0.713-3.100	0.290
NKp46 (MFI)	2174	0.822	0.919	0.442-1.914	0.822
**NKT-like cells**					
$CD16^+$ (%)	20.3	**0.000**	4.697	2.046-10.783	**0.000**
$CD158a^+$ (%)	5.1	0.068	1.969	0.937-4.136	0.074
$CD158b^+$ (%)	9.2	0.163	1.684	0.803-3.531	0.167
$NKG2A^+$ (%)	18.3	0.108	1.823	0.868-3.829	0.113
$NKG2A^+$ (MFI)	2472	0.780	1.110	0.533-2.315	0.780
$NKG2C^+$ (%)	10.7	0.462	1.314	0.634-2.726	0.463
$NKG2C^+$ (MFI)	1455	0.459	1.318	0.633-2.742	0.460
CD161 (MFI)	1630	0.682	0.858	0.413-1.784	0.682
$CD8^+$ (%)	75.9	0.442	1.332	0.640-2775	0.444
$CD8^+$ (MFI)	12934	0.523	1.268	0.611-2.632	0.524
DNAM-1 (MFI)	765	0.603	1.215	0.582-2.534	0.604
$NKG2D^+$ (%)	92.9	0.696	1.157	0.556-2.409	0.697
$NKG2D^+$ (MFI)	4704	0.394	1.385	0.653-2.940	0.396
NKp30 (MFI)	182	0.784	1.108	0.532-2.308	0.784
$NKp44^+$ (%)	1.2	0.221	1.591	0.751-3.370	0.225
NKp44 (MFI)	114	0.380	1.394	0.662-2.937	0.382

NKp46⁺ (%)	3.6	0.316	0.319	0.698-3.024	0.319
NKp46 (MFI)	53.4	0.563	1.240	0.598-2.572	0.564

[*]T cells were investigated in 25 CRC patients at risk of developing metastases
